# Scientists of Tomorrow/ *Cientistas do Amanhã* : a project to inspire, stimulate scientific thinking, and introduce scientific methodology for young students

**DOI:** 10.31744/einstein_journal/2023AE0622

**Published:** 2023-12-12

**Authors:** Érika Bevilaqua Rangel, André Luiz Teles e Silva, Érica Kássia de Sousa Vidal, Victória Tomaz, Caroline Mitiká Watanabe, Stephany Beyerstedt, Romário Oliveira de Sales, Eliezer Francisco de Santana, Rômulo Gonçalves Leão, Pedro Cancello, Thiago Pinheiro Arrais Aloia, Jaciele Conceição da Silva, Laudiceia Almeida, Letícia Bernardes de Oliveira, Luciana Cintra, Camila Hernandes, Lionel Fernel Gamarra, Eliseth Ribeiro Leão, Sidney Klajner, Luiz Vicente Rizzo

**Affiliations:** 1 Programa de Pós-Graduação Stricto Sensu em Ciências da Saúde Hospital Israelita Albert Einstein São Paulo SP Brazil Programa de Pós-Graduação Stricto Sensu em Ciências da Saúde , Hospital Israelita Albert Einstein , São Paulo , SP , Brazil .; 2 Faculdade Israelita de Ciências da Saúde Albert Einstein Hospital Israelita Albert Einstein São Paulo SP Brazil Faculdade Israelita de Ciências da Saúde Albert Einstein , Hospital Israelita Albert Einstein , São Paulo , SP , Brazil .; 3 Escola Municipal de Ensino Fundamental Professor Paulo Freire São Paulo SP Brazil Escola Municipal de Ensino Fundamental Professor Paulo Freire ; São Paulo , SP , Brazil .; 4 Hospital Israelita Albert Einstein São Paulo SP Brazil Hospital Israelita Albert Einstein , São Paulo , SP , Brazil .

**Keywords:** Education, Science, Researcher exchange, Students

## Abstract

The Scientists of Tomorrow/ *Cientistas do Amanhã* project is an immersive science training program developed by the Program of Post-Graduation in Health Sciences at *Hospital Israelita Albert Einstein.* This program was conducted in partnership with Volunteering and *Escola Municipal de Ensino Fundamental Professor Paulo Freire* in Paraisópolis, São Paulo, Brazil. The Scientists of Tomorrow Program comprised a short training period conducted in May 2022 involving 37 students, and a long training period from August to December 2022, which included 15 students. It aimed to popularize science through practical activities; transfer knowledge to young students; sensitize and guide them to pursue academic-scientific careers; reduce stereotypes about scientific work and scientists; and help students understand the social, political, and ethical roles of science within society. All activities were led by postgraduate students and professors from our postgraduate program, physicians, nurses, physiotherapists, biomedicals, and veterinarians from *Hospital Israelita Albert Einstein,* as well as medical students from *Faculdade Israelita de Ciências da Saúde Albert Einstein* . Activities in the short training included lectures on cinema and science, strategies to combat fake news, non-violent communication, innovation, design-thinking framework, and developing a scientific project. During the long training period, discussions were focused on nanotechnology, animal research, big data, bioinformatics, meditation, blood and bone marrow donation, telemedicine, sex and sexually-transmitted infections, rehabilitation, career opportunities, and scientific integrity. In addition, practical activities were further expanded using optical and confocal microscopy, cytometry, and basic concepts regarding the structure and function of living cells. The program also included the launching of the open-air outreach Education E-natureza activity, which turned students into ambassadors of nature. In conclusion, the Scientists of Tomorrow Program was innovative and enabled young students to learn that science is a collective activity that can enhance public health.

## Summary

In Brief

Rangel et al. enumerated the Scientists of Tomorrow/Cientistas do Amanhã program, an immersive science initiative conducted in collaboration with a public school. The program, which involved 15 students, aimed to promote science, share knowledge, inspire academic paths, and underscore societal impacts. Led by postgraduates, professors, and healthcare experts, the program included diverse lectures and practical laboratory activities.

## Abstract (key points)

Highlights

Every research endeavor commences with a fundamental question. Sharing of findings by researchers and students contributes toward the expansion of knowledge.

Teaching scientific methodology is a pivotal step in nurturing critical thinking skills.

Science permeates our daily lives and plays a crucial role in addressing societal issues.

## Graphical abstract

**Figure:**
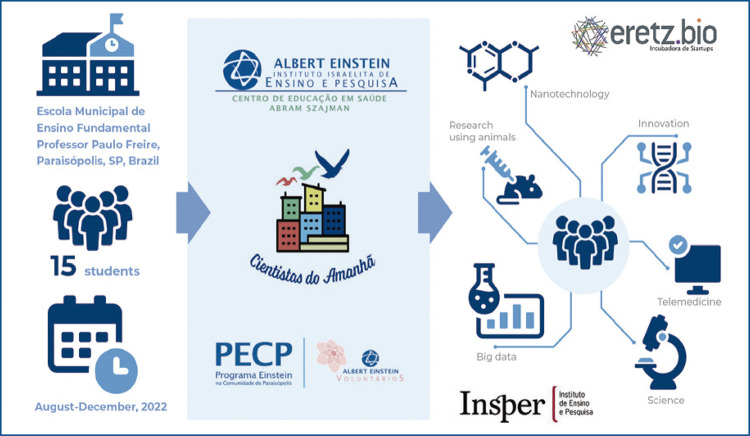

## INTRODUCTION

The Scientists of Tomorrow/ *Cientistas do Amanhã* project was created in 2022 at *Hospital Israelita Albert Einstein* (HIAE), São Paulo, SP, Brazil. This program is a collaboration with the *Stricto Sensu* Post-Graduation Program in Health Sciences at HIAE that included professors and post-graduate students (Master’s and Ph.D.) and the *Escola Municipal de Ensino Fundamental Professor Paulo Freire,* an elementary public school located in Paraisópolis, one of the largest slums in the city of São Paulo. This school is served by Albert Einstein volunteers who are part of our institutional program to help the underserved, called the Einstein Program in the Paraisópolis Community (PECP - *Programa Einstein na Comunidade Paraisópolis* ).

The main objective of the initiative was to provide an immersive experience in science for young students, starting from Grade 9. Practical activities focused on experimental and clinical scientific research laboratories along with tasks to encourage scientific thinking. We also aimed to stimulate scientific methodological thinking through different approaches and platforms to promote the understanding that science is a collective activity with the goal of improving knowledge and benefitting society overall.

When we launched the Scientists of Tomorrow project, specific objectives were defined and shared with other researchers and health professionals as follows:

1) Make scientific knowledge accessible to elementary education students at public schools and introduce science topics in their daily lives to transform their thinking; 2) Increase interaction between the university academic environment and public schools of elementary education offered by public schools; 3) Enhance learning conditions and encourage socialization of young people through social promotion and integration; 4) Introduce strategies to improve teaching and learning conditions aligned with local, regional, and global realities; and 5) Provide experiences and tools to showcase science as a collective activity.

The founding hypothesis aims to expose students to the construction of scientific thinking, including the formulation of hypotheses, addressing the hypotheses, and understanding how science benefits society. Accordingly, the program sought to foster the idea that science is a collective activity that allows everyone to participate.

## Description of the Scientists of Tomorrow/ *Cientistas do Amanhã* project: short immersive program

Initially, 37 students aged 14-15 from *Escola Municipal de Ensino Fundamental Professor Paulo Freire* participated in a short immersive training program in science from May 23-27, 2022, at the Albert Einstein Education and Research Center ( *Centro de Educação e Pesquisa Albert Einstein* ). The age group was selected based on the content covered in elementary school syllabus, ensuring that the students have already been exposed to science.

**Figure 1:**
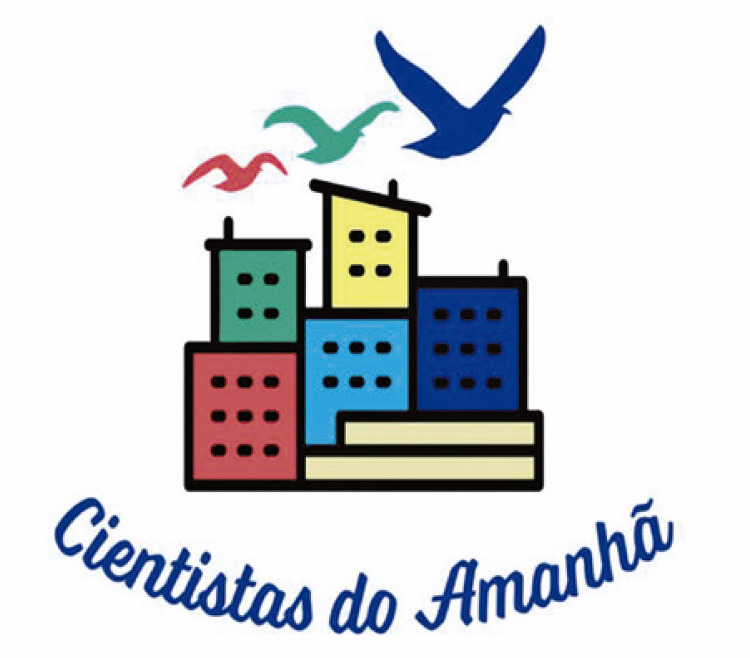
The slogan of Scientists of Tomorrow/ *Cientistas do Amanhã* Program. The project logo was created by Paulo Visintin, a designer and Master’s student from the Program of Post-Graduation *Stricto Sensu* in Health Sciences at HIAE

During the short program, the students received lab coats, T-shirts, and bottoms with the slogan Scientists of Tomorrow project ( Figure 1 ), and a badge with their names. They also received a certificate of participation in the project at the conclusion of training. Such materials may help sustain students’ involvement with the project even after its completion.

To learn more about students’ expectations and delineate actions for improving the project’s future editions, we conducted a satisfaction survey to obtain students’ feedback after each activity. The main questions were “What did you like the most?” and “Based on what you have seen today, how do you think this project will contribute to your life?”

Next, we provided a brief description of the short immersive science training program in May 2022 and the feedback provided by students ( Table 1 ):

**Table 1:** Feedback from students of the short immersive training program on science

1 ^st^ meeting, 05/23/2022: Focus on science and research
What did you like the most? *“From cinema and photography conversations, I liked the first photo and the first video or film performed in the world.” “I liked how they took pictures in olden days and the science behind cinema.” “I liked the explanations about cinema and being able to discover its origins.”* Based on what you saw today, how do you think this project will contribute to your life? *“Knowledge about cinema and photography and studies about space and knowing the relationship between cinema and science.” “Science helps people in different ways.” “I think I’m going to interpret movies, images, and so on.”*
**2 ^nd^ meeting, 05/24/2022: Combating fake news and adopting non-violent communication**
What did you like the most? *“The lecture on non-violent communication and knowing how communication can be violent sometimes.” “I liked the final part the most, where we wrote on a blackboard about non-violent communication words and fake news.” “I liked the fake news chat the most.”* Based on what you saw today, how do you think this project will contribute to your life? *“The importance of non-violent communication, because, in several of my speeches, I speak aggressively without the need for that.” “I can choose to use non-violent communication with my family and friends.” “As fake news spreads, don’t share them. I will talk to my family and friends about not sharing fake news as well.” “The question of knowing how to identify fake news and understanding the four steps of non-violent communication [observations, feelings, needs, and requests]. This information will help me evolve further, and I will be careful about what I’m going to do or repost, in terms of fake news, and avoid violent communication.”*
**3 ^rd^ meeting, 5/25/2022: Innovation focus**
What did you like the most? *“I enjoyed everything, Paulo’s lecture and the chat with Camila.” “Paulo’s lecture on how he managed to achieve what he is today.” “I learned about robotics, how to start a business, I understood more about finance.”* Based on what you saw today, how do you think this project will contribute to your life? *“Paulo’s teachings on informatics and technology will help me evolve in innovation.” “I’m going to imbibe this so that I don’t give up and will study further.” “The importance of studying. I arrived at this answer through the story of Paulo, owner of Hoobox, who spoke about the importance of studying in his career.” “I became curious about computer science and will investigate further on this topic.”*
**4 ^th^ meeting, 05/26/2022: Innovation focus**
What did you like the most? *“Understanding how design thinking works.” “The presentations of other students, the activities we have done today, and the conversations.” “The lecture and the idea of creating a product and presenting it.”* Based on what you saw today, how do you think this project will contribute to your life? *“It helped me improve my communication because I had to work in a group and convey my ideas.” “Knowing how to solve problems in society. Maybe I can work in this area in the future.” “Design thinking, because it’s interesting and helps us get organized.”*
**5 ^th^ meeting, 05/27/2022: Construction of a research project**
What did you like the most? *“Everything! Today was amazing! I loved making posters, the presentation, and the laboratory, I loved it!” “From working in a new group and observing the lab and seeing the root of the plant under the microscope.” “From presenting the piece, going to the lab, talking about COVID-19, and everything.”* Based on what you saw today, how do you think this project will contribute to your life? *“Everything, the laboratory made me think whether I should be a scientist.” “I learned that vaccines are safe and how they are produced.” “How the clinical picture works, because I learned how it works.” “Washing hands correctly.” “Everything I learned this week was incredible. I’m imbibing into my life everything I learned in Vila Mariana and Morumbi.”*

**Figures 2:**
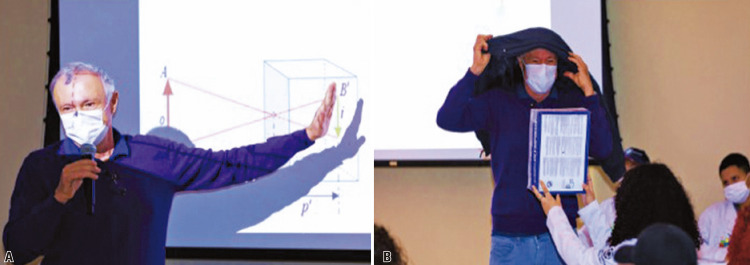
A-B. Science and its connection with cinema conducted by Prof Márcio Barreto

May 23 ^rd^ (Monday): students of the *Escola Municipal de Ensino Fundamental Professor Paulo Freire* were categorized into six groups. They had a welcome breakfast in an open and wooded area with postgraduate students from the *Stricto Sensu* Post-Graduate Program in Health Sciences from HIAE at the Albert Einstein Education and Research Center, Campus Cecília, and Abram Szajman. The elementary school students had the opportunity to have informal and leisurely conversations with young scientists from the post-graduate program. Next, all teams gathered to listen to an inspiring lecture regarding the importance of research in our lives and within society by Research Director Prof. Luiz Vicente Rizzo. The lecture was followed by a motivating talk titled “Science with Popcorn” by Prof. Márcio Barreto from the Faculty of Applied Sciences of the *Universidade Estadual de Campinas* (UNICAMP). His work explored how cinema could be associated with scientific proposals. This activity enabled students to understand how observations can help advance knowledge, such as when using a camera obscura, which was mimicked during the activity using a shoebox (Figures 2A-B). The students observed how light could enter a darkened area through a small aperture, and ultimately create a rudimentary image that was upside down on the wall facing the opening. In addition, the students learned how this knowledge contributed to the evolution of photography and filmmaking as we know it today.

May 24 ^th^ (Tuesday): combating fake news and adopting non-violent communication.

The spread of COVID-19 across the globe was accompanied by a tsunami of disinformation, misinformation, fake news, and conspiracy theories. ^( 1 )^ It undermined trust in global health institutions and science, as was observed in vaccine hesitancy. ^( 2 )^ This can be addressed by increasing the level of accurate information for society.

During the activity on “combating fake news,” the students listened to a talk on the following points: 1) an introduction to fake news and the role of the public; 2) a dynamic analysis of true news and identification of fake news; 3) techniques to determine whether the news is true or false, including access to Lupa agency, which is dedicated to checking the veracity of facts.

**Figures 3:**
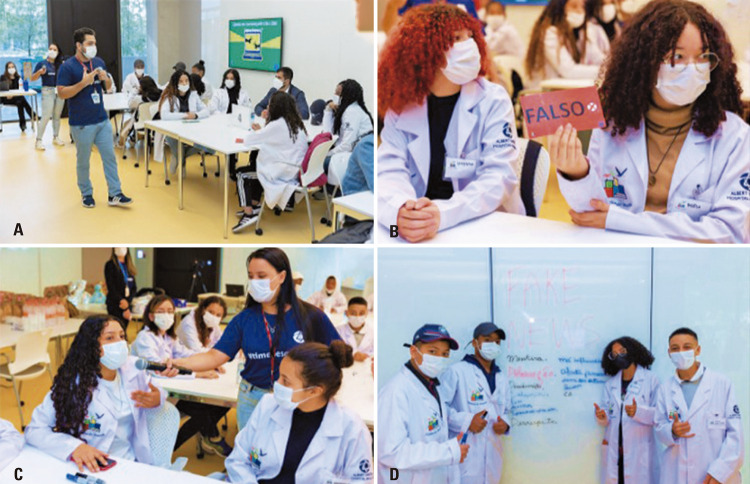
A-D. “How to combat fake news?” activity with post-graduate students André Teles and Érica Kássia Vidal

In the dynamic activity, we attempted to help students identify whether the news was factual or fake, and write down the meaning of fake news. Therefore, students in each group displayed a sign indicating whether the information was fake or true (Figures 3A-D). The group with the highest number of correct answers was declared the winner. Importantly, gamification is considered a framework that promotes transformative learning, which increases academic performance and overall motivation. ^( 3 )^ Therefore, ultimately, our goal was to make students aware of the negative impact of fake news, demonstrate how important it is to spread the truth, and to what extent it is possible to play this role with care and education. We had the perception that students decided not to pass on false information and chose news sources that they considered reputable, all of which would make a major difference. The students concluded that we can consider misinformation and disinformation in the same manner as the actual virus (SARS-CoV-2), given that both spread quickly.

In the activity on non-violent communication, Prof. Germana Barata from UNICAMP demonstrated the prevalence of violent communication in daily life. Students were able to provide examples and report conflicting situations. They also learned that non-violent communication methods involve four steps: 1) sharing observations without judgment, 2) expressing feelings, 3) connecting those feelings to needs, and 4) making requests from others.

**Figures 4:**
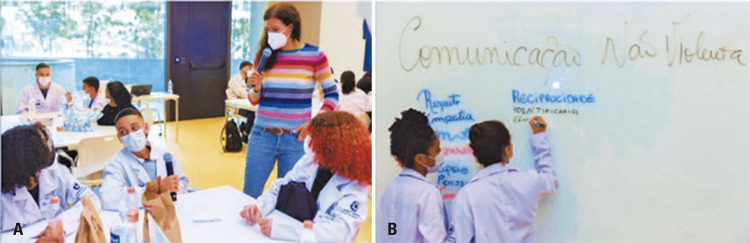
A-B. Non-violent communication activity with Prof. Germana Barata

In the practical activity conducted during the training, the students could write on a board about how to switch from violent to non-violent communication, and they also had the opportunity to read each other’s experiences (Figures 4A-B). Considering that non-violent communication could be a useful tool during the learning process, ^( 4 )^ the goal of the activity was to include students’ voices in the resolution and prevent conflicts to reduce violence, strengthen friendships, establish caring behaviors, and empower them as transformative agents within their communities.

**Figures 5:**
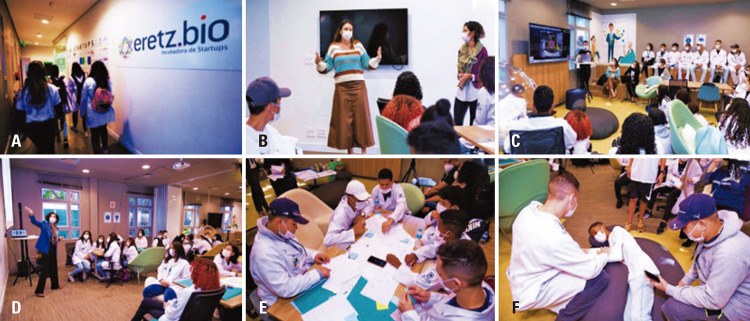
A-F. Innovation activities at Eretz.bio (A), including lectures of Camila Hernandes (B), Paulo Pinheiro (C), and Vivian Pereira de Brito (D), and design thinking activity with the development of the App “Locate Urgently” by students (E-F)

May 25 ^th^ (Wednesday): innovation at Eretz.bio:

Eretz.bio is an incubator for startups supported by HIAE ( Figure 5A ), which is dedicated to encouraging entrepreneurship to transform Brazilian health. The incubator is located in the Vila Mariana neighborhood in the city of São Paulo. The initial activity included a motivational lecture given by Camila Hernandes, Ph.D., an innovation manager at HIAE. In this lecture, students learned the role of science and innovation in the global health sector ( Figure 5B ). The students also attended a lecture with Paulo Pinheiro, Ph.D., co-founder and CEO of the startup HOOBOX Robotics, who narrated an inspiring story about building a startup, including challenges and opportunities ( Figure 5C ). HOOBOX Robotics is the world’s first health technology company to develop computer vision and facial recognition technologies in the healthcare sector. Their solutions focus on rehabilitation and therapies supported by technologies such as virtual reality and sensors. The students were inspired by Paulo’s story. They also visited some startups in Eretz.bio and had brunch with researchers and entrepreneurs, as part of relaxation and interaction.

May 26 ^th^ (Thursday): innovation at Eretz.bio: Techthon - Technology Marathon:

The students were categorized into six groups at Erezt.bio. Dynamic activity was conducted so that students could develop their innovation projects using the design-thinking framework under the supervision of Vivian Pereira de Brito ( Figure 5D ). Design thinking is a creative problem-solving framework to better understand challenges and generate solutions for health education. ^( 5 )^ This approach has been used to explore and address the challenges related to health systems. The workshop participants came up with ideas to improve access to healthcare units by risk-stratifying individuals in poor communities, incorporating community agents according to the number and severity of diseases, and establishing continuing educational programs to inform about prevention and adherence to treatment. One of the most amazing ideas was the creation of a mobile App, named “Locate urgently,” to promote interaction between communities, ambulances, and traffic and navigation Apps. The Apps would help identify the best route, either faster or safer, to enable people to reach the hospital, as poor communities usually do not have easy access to hospitals (Figures 5E-F). This idea was further discussed at Insper ( *Instituto de Ensino e Pesquis* a) one of the most important Brazilian higher-education institutions operating in the areas of business, economics, law, mechanical engineering, mechatronics engineering, computer engineering, and computer science. The students learned how to create the App, especially the challenges and opportunities involved.

**Figure 6:**
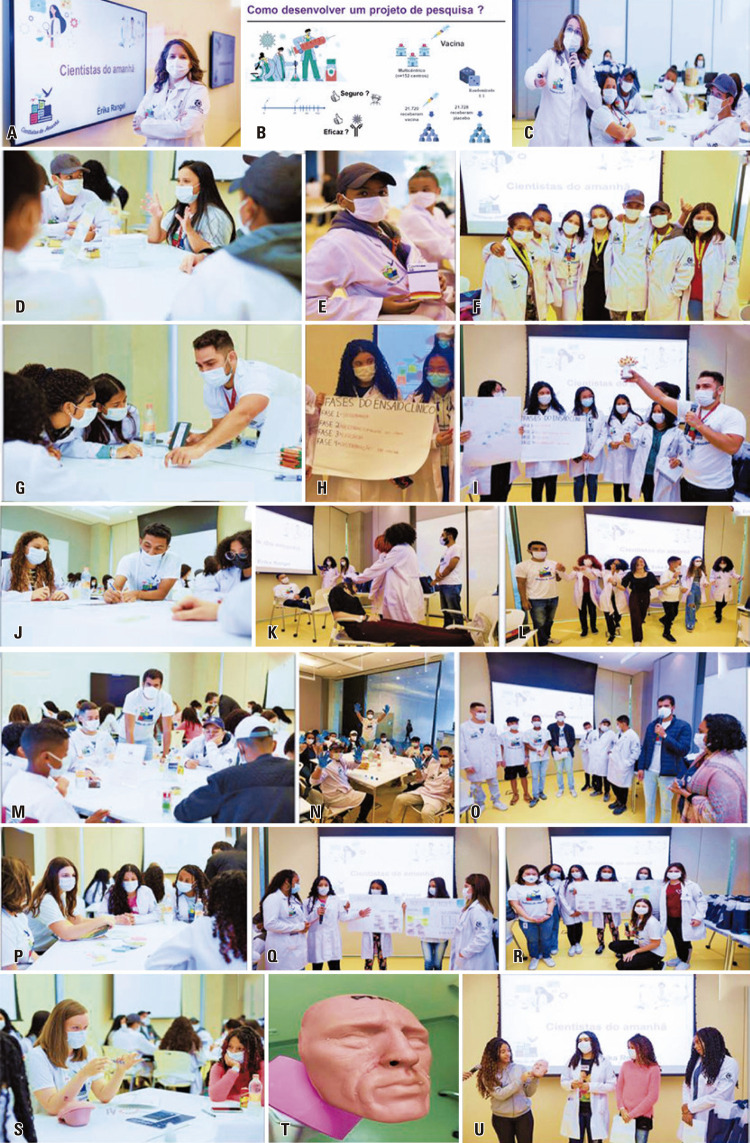
A-U. “How to design a scientific project?” activity. The main steps of scientific research in the COVID-19 setting were explained by Prof. Érika Rangel (A-C). Subsequently, the students were classified into groups to develop a COVID-19-related project along with post-graduate students. Group 1 from Érica Kássia Vidal developed a project based on a medication to treat COVID-19 (D-F) whereas Group 2 from André Teles developed a vaccine-based project (G-I). Group 3 from Romário Sales developed a clinical screening protocol to identify the main symptoms of COVID-19 (J-L), and Group 4 from Eliezer Santana worked on COVID-19 prevention, including how to wash hands correctly (M-O). Group 5 from Victória Tomaz and Caroline Mitiká developed a project to identify and treat post-COVID-19 syndrome (P-R), while Group 6 from Stephany Byerstedt worked on COVID-19 diagnosis, including nasal swab, and discussion of molecular techniques (S-U)

5) Friday 27 ^th^ (Friday): introducing students to the scientific method:

On the last day of the short immersive science training program, the students were encouraged to develop a research project on various topics, including COVID-19, at the Cecília and Abram Szajman campus of the Albert Einstein Teaching and Research Center. The activity was mentored by Prof. Érika Rangel, a medical researcher at HIAE, who gave an informative lecture on COVID-19. Her presentation included topics such as identification of SARS-CoV-2, its spread, prevention, clinical symptoms, laboratory diagnosis, and the role of science in reducing the COVID-19 pandemic burden worldwide (Figures 6A-C **)** . She highlighted the main steps in developing new drugs and vaccines, including preclinical (cell culture and small animals) and clinical experiments (the different phases of a clinical study, scientific methodology, and concerns regarding safety and efficacy).

After the lecture, the Scientists of Tomorrow students were classified into six groups and mentored by postgraduate students from HIAE. Considering that COVID-19 was the main theme, each group discussed the topic, developed a project based on scientific information, and prepared oral presentations. The goal was to give each group the opportunity to learn from each other’s project ideas (Figures 6D-U):

Group 1: Érica Kássia Vidal (Master’s student)-Theme: How to develop drugs to treat COVID-19?

Group 2: André Luiz Teles (Ph.D. student)—Theme: How to develop vaccines to combat COVID-19?

Group 3: Romário Oliveira de Sales (Ph.D. student)-Theme: What are the main symptoms of COVID-19 and how to risk-stratify patients?

Group 4: Eliezer Santana Júnior (Master’s graduate)- Theme: How can I contribute to prevent the spread of COVID-19?

Group 5: Victória Tomaz (Master’s graduate) and Caroline Mitiká Watanabe (Ph.D. student)-Theme: What is post-COVID syndrome and its impact?

Group 6: Stephany Beyerstedt (Master’s student)-Theme: How to diagnose COVID-19?

After a brainstorming session, the students came up with interesting projects demonstrating how and what they learned from the topics presented, that is, creativity, the choice of accurate information on science, employment of non-violent communication, practice of a design-thinking framework, and how to demonstrate motivation and enthusiasm. Moreover, the take-home message from the training program was that science is a collective activity that could improve public health. At the end of the session, it was clear that successful mentorship enhanced students’ knowledge of learning and development, as demonstrated in other studies. ^( 6 )^ To be a mentor is rewarding, and contributes to the improvement of personal and professional development. ^( 7 )^ That was the impression we all gained.

**Figures 7:**
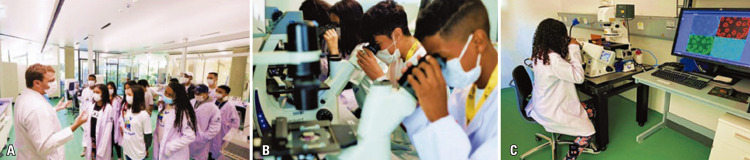
A-C. Prof. Dr. Geraldo Medeiros-Neto’s visit to the experimental biology laboratory at HIAE along with Thiago Aloia

The final activity included a guided tour by Thiago Pinheiro Arrais Aloia, Ph.D., a research assistant at HIAE in the Experimental Biology Research Laboratory. During the tour, the students observed cell structure using light and confocal microscopy ( Figure 7A -C). The following topics were discussed during the visit: “What does a scientist do in a laboratory?” “How does it work?” “What is the function of a microscope?” “Preparation of cells and tissues to be viewed under the microscope,” “Identification of the cell membrane, cytoplasm, and nucleus using the microscope,” and “Visualization of microscopic structures of the human body (cells, tissues, and organs).” This approach of combining education and science seems to be valuable in advancing knowledge of scientific ideas and promoting critical scientific thinking. ^( 8 )^

**Figure 8:**
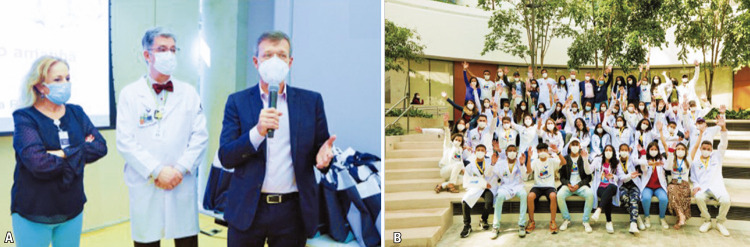
A-B. Closing ceremony with Telma Sobolh, Prof. Luiz Vicente Rizzo, Dr. Sidney Klajner, and the entire team

The closing ceremony of the project included a visit from Dr. Sidney Klajner, president of *Sociedade Beneficente Israelita Brasileira Albert Einstein* (SBIBAE), Prof. Luiz Vicente Rizzo, and Mrs. Telma Sobolh, volunteer-president at SBIBAE ( Figure 8A ). All the participants gathered for a group photo ( Figure 8B ).

Despite being a short immersive science training project, we observed that combining science and education paves the way for the development of critical thinking skills among young students.

## Description of the Scientists of Tomorrow/ *Cientistas do Amanhã* project: long immersive program

The second part of the program or the long immersive science training was held from September to December 2022, in which 15 students participated. They received a Scientific Initiation scholarship of approximately R$ 800.00 each per month, similar to the scholarships provided by public agencies in Brazil such as the Research Support Foundation of the State of São Paulo (FAPESP *- Fundação de Amparo à Pesquisa do Estado de São Paulo* ) and the National Council for Scientific and Technological Development (CNPq *- Conselho Nacional de Desenvolvimento Científico e Tecnológico* ). Additionally, the students received a lunch box during the training. Transportation to the campus was borne by the students, except on days when external visits were scheduled, where a chartered bus was provided by HIAE. The total cost of the program was approximately R$ 50,000.00.

The schedule included two activities per week (Monday and Tuesday) from 2pm to 5pm. All the activities were followed by postgraduate students from HIAE who were previously involved in the short immersive training program in May 2022. The program also involved communication through WhatsApp groups and social media platforms, mainly Instagram, which connected them with post-graduate students and professors. All postgraduate students and healthcare professionals of HIAE volunteered to participate in the program.

The long immersive training program on science was based on a partnership between basic research, clinical research, innovation, the startup incubator Erezt.bio supported by HIAE, and hospital care. The second part of the program involved a multidisciplinary approach ( Table 2 ).

**Table 2:** Long immersive training program on science

September 2022
05/09: Presentation of scientific pre-initiation program, highlighting benefits and tasks. Included a team of post-graduate students (André Teles, Érica Kássia Vidal, Victória Tomaz, Caroline Mitiká, Eliezer Santana, Stephany Beyerstedt, and Romario Sales) and Prof. Érika Rangel, as well as activities with Alessandro Marins and Thiago Aloia (laboratory activities).
06/09: Experimental biology activities with Alessandro Marins and Thiago Aloia.
12/09: Addressing anxiety and meditation with Prof. Elisa Kozasa.
19/09: Big data with Prof. Helder Nakaya and project consultant Joselisa Peres Queiroz de Paiva.
20/09: Bioinformatics with Victória Tomaz.
26/09: CETEC ( *Centro de Experimentação e Treinamento em Cirurgia Einstein* ): Large and small animal facility. Learning the regulatory aspects of animal research, understanding animal models for studying human diseases, and performance of small procedures (injection of saline solution in rodents, for example).
27/09: CETEC ( *Centro de Experimentação e Treinamento em Cirurgia Einstein* ): Small and large animal facility. Learning the key aspects of research involving large animals and introduction to the robotic da Vinci Surgical System.
**October 2022**
03/10: Academic trajectory with Prof. Juliana Magdalon.
04/10: Visit to magnetic resonance imaging, report rooms, and research involving this structure with Prof. Ronaldo Baroni.
10/10: Visit to the Blood Bank and Cell Therapy Laboratory with Leticia Taba, Kelen Cristina Alvarez, and Dr. Andrea Kondo.
11/10: Experimental biology laboratory activity with Thiago Aloia.
17/10: Visit to HIAE with Hospitality and Rehabilitation professionals.
18/10: Launching of E-natureza Ambassadors Program with Prof. Eliseth Leão.
24/10: Flow Cytometry Activity-Training cells for variation with Amanda Figueiredo and post-graduate students Victória Tomaz and Caroline Mitiká.
25/10: Non-communicable chronic common diseases: Obesity and Diabetes (Prof. Erika Rangel); Sleep (Prof. Patrícia Aguiar); Aging (Prof. Edson Amaro).
31/10: Lecture about how innovation can help us find solutions for society.
**November 2022**
01/11: Cinema “Reports of the Pandemic” with Prof. Luiz Vicente Rizzo and Prof. Érika Rangel at Kleinberg Auditorium, HIAE.
07/11: Nanotechnology with Prof. Lionel Gamarra, and post-graduate students Arielly da Hora and Gabriel Nery.
08/11: Nanotechnology with Prof. Lionel Gamarra, and post-graduate students Arielly da Hora and Gabriel Nery.
21/11: Lecture by Renata Lopes from PECP, Career and Integrity with Deyse Noronha from the Scientific Integrity office of HIAE.
22/11: Lecture on heart, lung, and liver by medical students of FICSAE and visit to the anatomy laboratory with Rômulo Leão and medical students of FICSAE.
28/11: Activity on DNA extraction in the experimental laboratory with the post-graduate students Andre Teles and Erica Kássia.
29/11: Conversations surrounding gender and pregnancy with Dr. Saulo Vito Ciasca, followed by STIs (sexually-transmitted infections) with Dr. André Mario Doi, including Rômulo Leão and other medical students from FICSAE.
**December 2022**
02/12: Visit to Burle Marx Park with Prof. Eliseth Leão.
05/12: Group activities: All graduate students presented their projects.
06/12: Closing ceremony: All graduate students received a certificate of participation.

The sequence of activities was chosen to promote an association between bench and bedside applications, emphasizing the program’s contribution toward prevention, diagnosis, and disease treatment. Additionally, the program aimed to provide students with a broad view of health careers and opportunities to plan their next step as students, thereby enhancing their career development.

In September 2022, the students participated in several practical activities, including microscopy and cytometry analyses, in the experimental biology laboratory of Prof. Dr. Geraldo Medeiros-Neto, Albert Einstein Education and Research Center. For the microscopy activity, Thiago Pinheiro Arrais Aloia proposed a theoretical-practical activity consisting of lightly scraping the internal mucosa of the cheek using disposable spatulas, flexible cotton swabs, or wooden sticks and preparing a smear by spreading it over a glass slide. Students were then challenged to draw what they observed under the microscope and identify the structures that were visualized. The students also answered a quiz: “What is the function of 70% alcohol during the preparation of material for observation?” “Was it possible to observe bacteria (prokaryotic cells) in the smear? Comment on that,” “What is the format of the oral mucosa cell?” “What are the major differences between prokaryotic and eukaryotic cells?” and “What did you learn from today’s activity?”

The proposed activity allowed putting into practice concepts that worked in the classroom, breaking down barriers, and providing an intimate education-science interaction. The students were enthusiastic, curious, and impressed with their experience. The fact that they studied the cells taken from the mouth in the laboratory made the activity fun and, simultaneously, contributed to learning about the basic characteristics and features of a eukaryotic cell viewed under an optical microscope.

**Figures 9:**
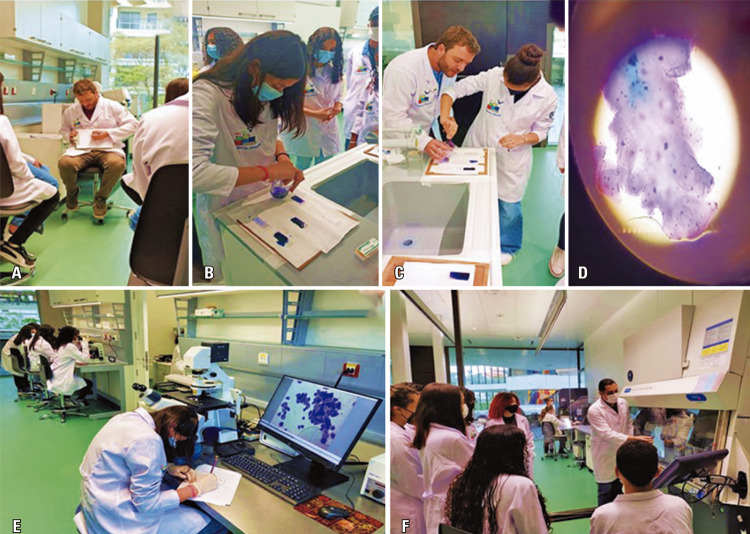
A-F. Experimental biology laboratory activity. Visualization of epithelial cells from cheek mucosa using a light microscope under Thiago Aloia supervision (A-E). Demonstration of the principles of flow cytometry (lasers, filters, and hydrodynamics) with Alessandro Marins based on theories of optics and wavelength that students were able to correlate to school science topics (F)

As illustrated in figures 9A-E, the results were extremely positive, arousing students’ interest in science and health. The students also learned from cytometry analysis that cells could be labeled with fluorescent dyes and identified using different wavelengths from the laser of a cytometer, which could be useful for the diagnosis and follow-up of various diseases ( Figure 9F ). This activity was supervised by Alessandro Marins. In September 2022, the students participated in meditation activities, learned about big data, received basic information about research involving small and big animals, and observed the robotic da Vinci surgical systems (Si, X, and Xi; Sunnyvale, California, USA). They also had the opportunity to handle small rodents and subcutaneously inject saline solution. Importantly, they learned that there are regulatory rules established by the National Council for the Control of Animal Experiments (Concea - *Conselho Nacional de Controle de Experimentação Animal* ) for conducting research using animals in Brazil. We also highlighted that the 4R’s (responsibility, refinement, replacement, and reduction) are key to good research practices. ^( 9 )^ The students learned that knowledge gained through experiments is beneficial to society, while researchers have a moral duty to minimize harm to small and large animals.

The bioinformatics activity, organized by postgraduate student Victória Tomaz, aimed to demonstrate the importance of information technology in the health sector and its applicability in detecting SARS-CoV-2. The activity involved the evaluation of DNA, RNA, and protein sequences; the study of three-dimensional structures of molecules; and the visualization and analysis of images and biological signals. In the first part of the activity, some basic concepts were explained, such as the difference between DNA and RNA, what is a virus, what is the structure of a virus, how to perform sequencing analyses for the detection of genetic material, what is SARS-CoV-2, what are the variants of SARS-CoV-2, and the laboratory method used to determine if a patient is infected. In the second part, the bioinformatician Erick Dorlass participated to briefly explain bioinformatics and the “codes” that we use extensively in informatics. During the activity, each student had a computer equipped with a bioinformatics pipeline. In this pipeline, we taught basic commands such as mathematical calculations and how to demand specific functions. As part of a challenge in the third and final part of the class, each student randomly selected a sample from a patient infected with SARS-CoV-2. Each patient had different symptoms resulting from infection with different viral variants. We helped the students perform the bioinformatics “code” of calling variants for the samples of these patients and in the end they had to identify the SARS-CoV-2 variant of each patient. The students said, “I learned that, at the moment, we are not so different from a computer, because just as it learns and teaches, we also learn and teach,” and “I learned how to configure artificial intelligence through a robotic language (Python) and that this language will help us identify SARS-CoV-2 variants.”

At the end of September, the group of six Scientists of Tomorrow students that conceived the App “Locate Urgently” visited INSPER after contacting Mr. Irineu Gianesi. The students learned App creation from Mrs. Paulina Achurra. Two noon sessions were dedicated to the knowledge of the process of creating the App when some INSPER students visited to narrate their experiences. They were able to interview the firefighters by asking questions about the construction of the App and verifying its feasibility. They visited some of the “FAB LAB” rooms and scrutinized how things work. Their commitment revealed that they could experience the creation, process, and prototyping of an App. We recorded the testimony of Gustavo Ramos, one of the Scientists of Tomorrow students: “My experience of being able to go to INSPER with a project done by me and my colleagues was great, it was very gratifying to know that through an idea that we only debated, instructors were able to provide us with such a good activity. I managed to learn lessons for my whole life, knowing that an idea can sometimes be useless just because of lack of trust in talking to someone about it, but ultimately, it ended up being impressive. For me, it was great to have the opportunity to experience this activity, which was so different from our daily routine-I mean, from our routine on the periphery of our city-we can observe a different life that we mostly see in movies or through reports from friends, colleagues, or family members about the university environment.” Eduardo Camilo commented, “What did you learn at INSPER?” Answer: “About attitudes in life, why we do, what is the benefit, what will change. That part of knowing the reasons for things.” “Based on your learning, how do you think this knowledge will contribute to your life and why?” Answer: “To understand how to go deeper into issues and knowing why it was done, what has changed, and what will change. This is particularly useful for choosing a subject and paying attention to details.”

In October 2022, the students completed the hands-on activities in an experimental biology laboratory using a microscope and cytometry facilities. Victória Tomaz and Caroline Mitiká Watanabe, two post-graduate students from HIAE, developed a practical activity at a cytometry facility. The purpose of this activity was to teach students the basic principles of cell biology, such as cell size, cell complexity, and different types of defense cells (lymphocytes, monocytes, and granulocytes). Before commencing the practical activity, the students learned about immune cells and were introduced to wavelength-based methodology. During the practical class, the students themselves performed the activity; a high point of this activity was that it brought about engagement in the whole class. Students received instructions on Personal Protective Equipment (PPE) and good laboratory practices to avoid setbacks. Subsequently, the students marked the sample with previously-presented monoclonal antibodies, and the sample was acquired using a cytometer. All the equipment were presented so that the students could understand the different wavelengths, and most of the group managed to associate lasers and filters (optical lenses) with wavelength concepts. Interestingly, one of the students could not see the device’s ultraviolet (UV) laser. The group hypothesized that this could be due to the UV protection of his glasses. When the students understood how UV protection of glasses worked, we briefly tested a pair of glasses with UV-free and non-UV-free lenses to prove that the hypothesis was correct. At that moment, they were ecstatic about the discovery, and the experience certainly fixed their theoretical knowledge. That was fantastic!

**Figures 10:**
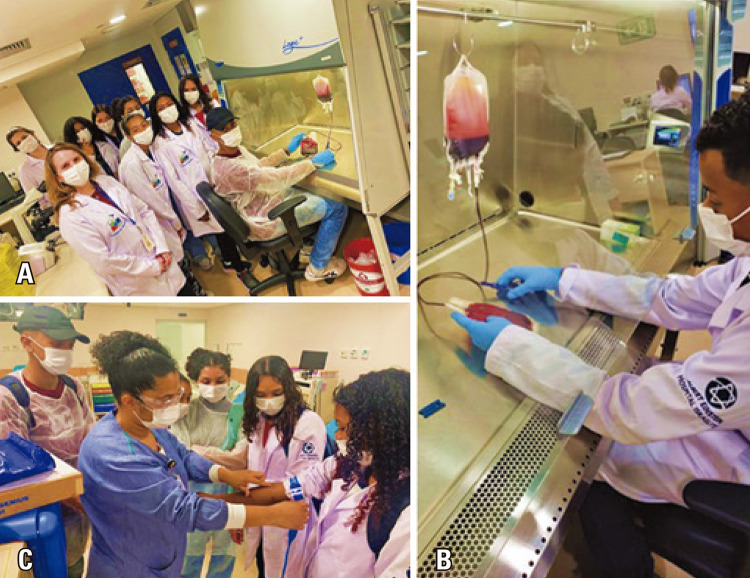
A-C. Visit to the blood bank at HIAE

The students also visited other clinical facilities at HIAE, including an imaging unit, blood bank, cell therapy, hospitality, and rehabilitation. They learned how blood and its products are stored and the importance of bone marrow and blood donation (Figures 10A-C). They also wrote down on the feedback form: “Now, I know that some blood types are rare and that blood and bone marrow donation can save lives!” I want to be a blood donor when I turn 16.”

The students listened to the academic trajectories at FICSAE by Prof. Juliana Magdalon, and medical and nursing students who are supported by scholarships. Through clinical research, they had learned about obesity ^( 10 )^ and sleep disorders. ^( 11 )^ In addition, the students listened to a talk about how sleep disorders are connected to obesity and aging ^( 12 )^ and how all information can be analyzed using the big data approach. Moreover, the implementation of the interprofessional learning approach enabled the perception that the most difficult challenges could be reframed as opportunities for change. ^( 13 )^ This reframing was clear when students, categorized into groups and through writing on the board, proposed changes in the health system in an attempt to reduce the obesity burden.

**Figures 11:**
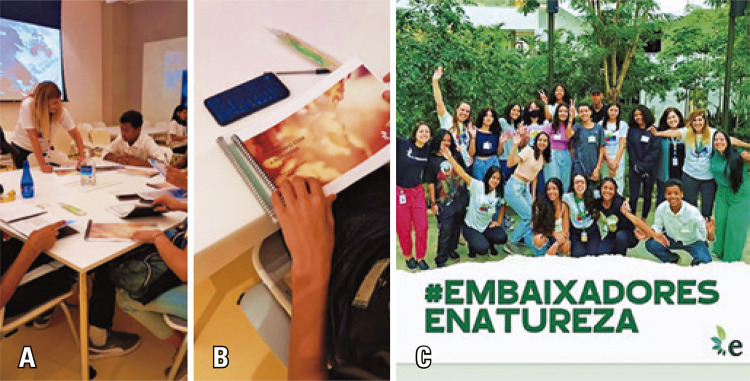
A-C. The launch of the e-Nature Ambassadors Program by Prof. Eliseth Leão

In mid-October, the e-Nature Ambassadors program was launched by Prof. Eliseth Leão and Letícia Bernardes de Oliveira (Figures 11A-C). Subsequently, the “e-Nature: Interdisciplinary studies on connection with nature, health and well-being” was presented to the Scientists of Tomorrow students. All participants were appointed as e-Nature Ambassadors so that, in addition to learning about the importance of connecting with nature and its benefits for human health and well-being, we also suggested that everyone share information with their family and friends. The goal was to encourage more people to connect with nature through various means.

For engagement, each participant received the Nature Inspiration Guide and an Eco-challenge Guide. Adherence was immediate, and everyone included in their social media profiles turned to be e-Nature Ambassadors and followed the project’s Instagram@umtempocomenatureza. They began to engage and share posts, stories, and videos about nature by tagging the projects.

The activity helped us to observe students’ engagement and generate data. Our plan is to recognize, after a few months, the most engaged student to attend the II International Symposium on Nature and Health at Einstein and share his/her experience. The research team will also donate books to other students who participate as e-Nature Ambassadors.

During the activity, one of the students, in addition to standing out for his project profile, created a profile on his own on Instagram @enatureza_embaixadores. To acknowledge his initiative, he and the students with the largest number of interactions were invited to participate with more experienced researchers for the book chapter “Relationship between human being-nature and social networks” that will be published in the book “ *Natureza, Clima e Saúde Pública* ” (Nature, Climate, and Public Health). The book will be published in Portuguese by Editora dos Editores and HIAE. These students were responsible for 45% of the nearly 200 interactions on Instagram. This interaction will help Scientists of Tomorrow students enhance their knowledge about the construction of a scientific project in a team and exchange experiences.

At the end of the face-to-face class (preceding the field practice), each participant was asked to utter two words. The first word defined their perception of the class. Some definitions are as follows: “liberating, unforgettable, incredible, wonderful, perfect, exciting, exceptional, splendid, amazing, differentiated, grandiose, great, influenceable, unique/special, comforting, purpose, reflective, and gratifying.” The second word concerned how the students felt when the class concluded. The students mentioned “anxious, curious, extremely happy, happy, proud, grateful, nervous, thrilled, excited, enthusiastic, euphoric, important, and impressed.”

The students also provided testimonies. One said “I will encourage people around me to take better care of nature and try to increase contact with nature”; another mentioned “It was good to know in depth how nature helps with many things in medicine, including depression and anxiety.”

**Figures 12:**
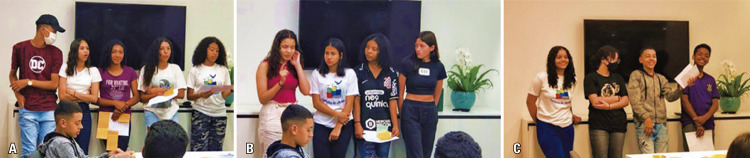
A-C. Design-thinking framework: “How to develop a scientific project?”

At the end of October, the students presented chronic disease-based projects in areas such as kidney and cardiovascular diseases, and stroke. These projects combined all the information about clinical practice, cell and molecular biology, logistics in the health system, and prevention. They also proposed innovative solutions through a design-thinking approach implemented by Eretz.bio (Figures 12A-C). This project-based learning methodology contributed to reflections on the health practices of students and allowed alternatives to transform them and their reality, as previously described by the pedagogical method proposed by Educator Paulo Freire ^( 14 )^ in an innovative setting. At that time, it came to our attention that the students were mature, critical, and capable of constructing a scientific project based on society’s demands. One of the students stated, “I enjoyed figuring out how to look for solutions to real problems,” another said “We need research to solve problems,” and the third reported, “I learned that knowing how to do projects will even help me organize myself.”

In November, we gathered to watch the movie *Portraits of a Pandemic* (“ *Retratos de uma pandemia* ”). This included a series of videos launched by HIAE regarding multiple efforts made to combat COVID-19, particularly using a multi-professional approach (https://globoplay.globo.com/retratos-de-uma-pandemia/t/SNFSGhhDR2/).

**Figures 13:**
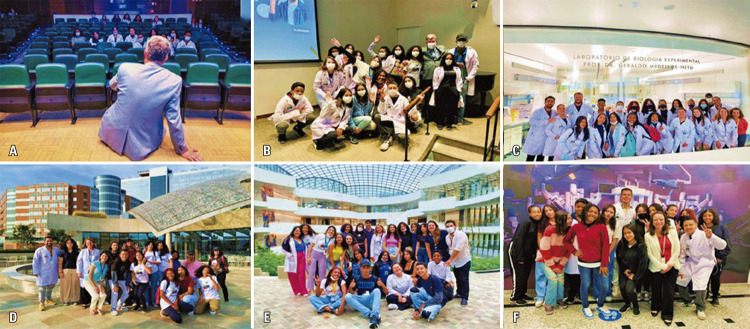
A-E. Moments of relaxation and interaction. Cinema session “ *Portraits of a Pandemic* ” with Prof. Rizzo at Kleinberg Auditorium at HIAE (A-B). *Faculdade Israelita de Ciências da Saúde Albert Einstein* (FICSAE), Campus Cecília & Abram Szajman (C-E). *Centro de Experimentação e Treinamento em Cirurgia Einstein* (CETEC) (F)

The students had a chat with Prof. Luiz Vicene Rizzo, and subsequently, they were impressed by how HIAE organized a support structure to treat patients from public health settings, and how professionals worked together to save lives. One of the students said “Thank you for showing the reality we did not know,” while another said, “To understand how other people have suffered in different ways during the pandemic and the need to adapt to different situations.” In figures 13A-B, we captured their moments of fruitful interaction with the Scientists of Tomorrow students. Several other moments of relaxation with the students and the HIAE team were captured using our lens (Figures 13C-E).

**Figures 14:**
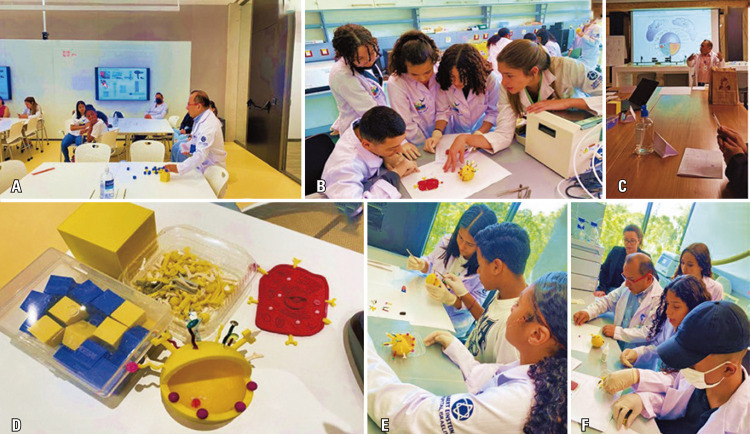
A-F. Nanotechnology activities supervised by Prof. Lionel Gamarra

Next, the students learned about nanotechnology from Prof. Lionel Fernel Gamarra, who addressed several aspects of nanobiotechnology, mainly those applied to the health sciences. This theoretical-practical class began with a presentation to help students understand the world of nanotechnology and relate it to their everyday activities. After a deductive explanation, students were able to understand the nanometric scale. These dimensions are related to different cells, and how they interact depends on the physico-chemical properties of nanoparticles, such as the area/volume ratio. Experimentally, 3D-printed pieces of magic cubes and samples of fine and coarse salt were used. After defining the properties of nanoparticles, students learned that it is possible to work with several common drugs, where the concentration is not the only indicator to determine effectiveness, but also the area/surface ratio of the drug components. Finally, Prof. Lionel Fernel Gamarra demonstrated how the interaction of nanoparticles with cells occurs. Accordingly, the students assembled a functionalized nanoparticle, and then assembled an animal cell with all the elements (Figures 14A-F). After assembling the nanoparticles and cells, the interaction between these elements and different applications were demonstrated. One of the students stated, “I enjoyed learning about nanometer and how nanotechnology is presented in everyday life.”

Scientists of Tomorrow students participated in various practical activities in the cytometry facility and learned about career opportunities from Mrs. Renata Garcia Lopez Perone at *Programa Einstein na Comunidade Paraisópolis* (PECP), a partnership between HIAE and the Paraisópolis community that focuses on developing educational activities. Students had the opportunity to listen to a talk on scientific integrity, which contributed to generating reliable information by Mrs. Deyse Noronha da Silva, who previously lived in the Paraisópolis community and is now working at HIAE.

The students also had the opportunity to improve their knowledge of the cardiovascular, respiratory, and hepatic systems in a dialogic class by two medical students from FICSAE. They were encouraged to reflect on how anatomical and physiological concepts apply to their daily lives. The students were constantly asked by the presenters to provide their opinions and points of view on the discussion. This dialogical approach, in which knowledge is constructed through dialogue between subjects, is one of the main characteristics of Paulo Freire’s pedagogy and one of the bases of Team-Based Learning, which is adopted in medical courses at FICSAE.

After the class, the students were led to a guided exhibition of synthetic and real pieces from the studied systems, including histology slides and anatomical pieces of pathologies prevalent in Brazil, such as Chagas cardiomyopathy, bronchopneumonia, and cirrhotic liver. Participants were classified into groups of five and were accompanied by two students from medicine, nursing, or physiotherapy courses at FICSAE. The students were guided through three stations: compromised lungs, heart, and liver.

They had the opportunity to explore each station for 15 minutes, and had the opportunity to tap into a comprehensive and personalized approach.

This activity was especially important for introducing students to pathology concepts through the observation of real human organs with some of the prevalent pathologies in Brazil. This activity provided a unique opportunity for both Scientist of Tomorrow and FICSAE students to develop their critical thinking and scientific reasoning skills. This approach is consistent with Paulo Freire’s educational theory. He argued that education should be a liberating and transformative practice where students are not merely receivers of information, but actively participate in the learning process, building their knowledge through dialogue and critical reflection of the world around them. ^( 15 )^ Therefore, the interaction of Scientists of Tomorrow students with medical students generated an opportunity to exchange knowledge and ideas in an experience that certainly contributed to both personal and academic development.

Next, Scientists of Tomorrow students participated in an activity based on genetics that was coordinated by post-graduate students André Teles and Érica Kássia Vidal. The study of genetics can often be considered complex and difficult for students to understand; therefore the main objective of this class was to demonstrate, using practical and simple examples, that genetics can be included in these students’ daily lives.

**Figures 15:**
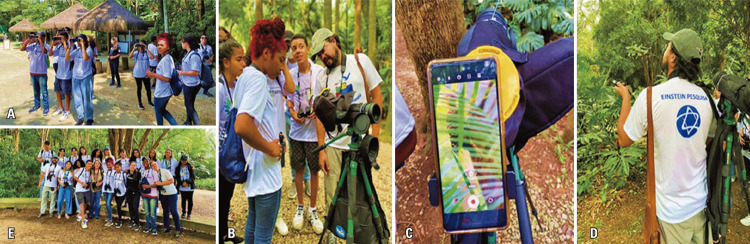
A-C. Activities on understanding basic concepts in genetics

During the class, participants had the opportunity to visualize chromosomes under a microscope and the assembly of karyotypes. This activity used differently-colored strings representing the chromosomes (each pair of the 23 chromosomes was colored differently) inside two balloons (the largest one represented the cell membrane and cytoplasm, and the smallest one, inserted inside the largest one, represented the nuclear membrane and nucleus, whereas the colored strings located inside the smallest balloon represented the chromosomes). Therefore, we sought a methodology that would facilitate students’ learning, enable the discussion of the importance of cell structures, phases of cell division, and study of chromosomal anomalies such as Down’s Syndrome (a group that has three chromosomes at position 21, indicating trisomy 21 chromosomes. Students had to figure out the explanation) (Figures 15A-C). We were able to motivate students throughout the class by bringing practical examples from everyday life, thus facilitating learning.

It is important to note that as the students had already learned the theoretical bases of genetics in previous classes, we anticipate that practical classes on DNA using simple and low-cost materials, including the extraction of genetic material from fruits such as kiwi, might improve students’ comprehension of more advanced concepts in genetics.

Next, the students had the opportunity to discuss the actual themes of sexuality, sexually-transmitted infections, pregnancy, and gender with Dr. Saulo Vito Ciasca, a psychiatrist with expertise in adolescent health. The students received a link from which they could answer questions anonymously about these themes. The questions were promptly answered, which contributed to breaking down taboos. Dr. Ciasca adopted a definition of sex that refers to four closely-interrelated axes: biological sex, gender identity, gender expression, and affective sexual orientation. In addition, students learned the importance of respect, regardless of gender or affective sexual orientation choices. One of Dr. Ciasca’s most important take-home messages concerned initiating an active sex life. “When you cannot say ‘NO,’ it is not okay.” Therefore, it is important to emphasize that we must say whether you agree or disagree with something, and that the decision must be respected.

Next, the students attended a short lecture with Dr. André Mario Doi about bacteria, viruses, and parasites, and how they cause diseases. They watched educational videos on these topics and visited the clinical laboratory at HIAE. The students observed the slides under a microscope with diplococci gram-positive bacteria that occurred in pairs in the case of *Neisseria gonorrhoeae* infection. They also observed different bacteria growing in a petri dish, laboratory tests performed on blood and stool, including a case of pork tapeworm infection by *Taenia solium* .

**Figure 16:**
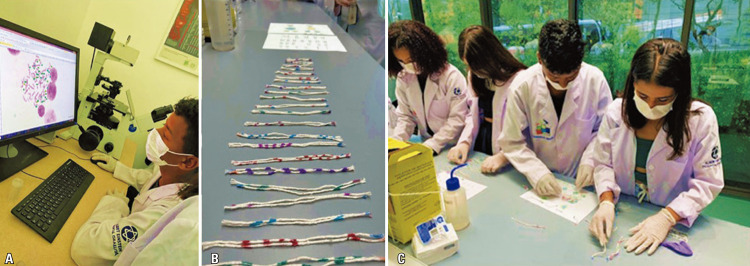
A-E. The open-air outreach education e-Nature activity at Burle Marx Park

In December 2022, a second face-to-face meeting of e-Nature Ambassadors was conducted at Burle Marx Park with Luciano Moreira Lima, an ornithologist from Butantan Institute, and a team of nurses, led by Prof. Eliseth Leão, to observe birds and learn about the fauna and flora of the city of São Paulo, as well as to reflect on their health and nature (Figures 16 A-E). The e-Nature research group has been available to share materials on the subject, and we have left the communication channels open for any ideas from students to increase green areas in their neighborhood.

Importantly, the program made two main adaptations. The first involved a partnership with INSPER to develop an App aiming to address the urgent arrival of ambulances to serve people living in Paraisópolis (“Locate Urgently”). Although the App was not developed because of the discovery of a similar existing App, students had the opportunity to learn the main steps involved in developing an innovative product.

The second adaptation involved a partnership with the e-Nature Program, coordinated by Prof. Eliseth Leão. During data collection, the students had the opportunity to write a book chapter and observe the development of a scientific project titled “Assessing the effectiveness of a nature-based multicomponent intervention for well-being and relationship with nature in different natural areas: a randomized clinical trial.”

Our study has some limitations. To address the Hawthorne Effect, where people change their behavior when they are aware they are being observed, we took measures to reduce the novelty effect of being observed by providing students with detailed information about the study without disclosing specific research goals. Additionally, we implemented a naturalistic observation approach, conducting observations in a natural setting ( *e.g.,* the e-Nature Program), allowing participants to become accustomed to the researcher’s presence and reducing the impact of the Hawthorne Effect over time. To address researcher bias, we developed a clear research protocol for group activities in the laboratory and classroom, providing specific research questions and procedures, thereby avoiding subjective interpretations. We also applied pre- and post-tests to objectively document students’ learning curves and capture their real perceptions. Moreover, researchers engaged in constant communication, fostering reflexivity in topics. This allowed them to continuously reflect on their biases and preconceptions throughout the study, acknowledging their potential influence on data collection and interpretation.

The main challenges involved measuring the impact of the training on students’ careers as they transitioned from elementary to high school in different locations. To enhance knowledge of scientific methodology and critical scientific thinking, we proposed implementing a Scientific Initiation Program with a longer duration ( *e.g* ., 1-year) for outstanding students. However, obtaining feedback on whether novel knowledge influences their community remains a challenge. Additionally, the selection process for the Scientific Initiation Program did not include a comprehensive evaluation of specific skills, such as communication, interpersonal abilities, learning/adaptability, teamwork, self-management, organizational skills, computer proficiency, problem-solving, and open-mindedness. Accordingly, during certain activities, such as teamwork training, some students preferred to work alone or in pairs, while others assumed leadership roles, speaking, and making presentations more than their peers.

Additionally, as expected, activities with limited practical or interactive aspects did not motivate students. Based on this observation, we adjusted the format of activities in subsequent editions to include more balanced practical tasks.

In conclusion, the Scientists of Tomorrow/ *Cientistas do Amanhã* project intended to popularize science, bringing it into the everyday life of adolescents, as well as share knowledge. By acquiring this knowledge, students can fight denialism and fake news. In addition, the information provided may encourage young students to pursue academic-scientific careers and develop the ability to practically apply scientific methodology.

Therefore, we aimed to transform lives through science and develop essential skills. This project will enable students to become important actors in critical thinking and disseminate scientific information within their communities. Ultimately, the project demonstrated how science is a collective activity that paves the way for the future of our country.
